# Role of Thylakoid Protein Phosphorylation in Energy-Dependent Quenching of Chlorophyll Fluorescence in Rice Plants

**DOI:** 10.3390/ijms22157978

**Published:** 2021-07-26

**Authors:** Aynura Pashayeva, Guangxi Wu, Irada Huseynova, Choon-Hwan Lee, Ismayil S. Zulfugarov

**Affiliations:** 1 Institute of Molecular Biology and Biotechnologies, Azerbaijan National Academy of Sciences, 11 Izzat Nabiyev Str., Baku AZ 1073, Azerbaijan; aynurapashayeva@gmail.com (A.P.); i_guseinova@mail.ru (I.H.); 2 Department of Integrated Biological Science, Department of Molecular Biology, Pusan National University, Busan 46241, Korea; guangxiwu1@gmail.com

**Keywords:** Lhcb, non-photochemical quenching, PsbS, phosphorylation, photosynthesis, rice, thylakoid membrane

## Abstract

Under natural environments, light quality and quantity are extremely varied. To respond and acclimate to such changes, plants have developed a multiplicity of molecular regulatory mechanisms. Non-photochemical quenching of chlorophyll fluorescence (NPQ) and thylakoid protein phosphorylation are two mechanisms that protect vascular plants. To clarify the role of thylakoid protein phosphorylation in energy-dependent quenching of chlorophyll fluorescence (qE) in rice plants, we used a direct Western blot assay after BN-PAGE to detect all phosphoproteins by P-Thr antibody as well as by P-Lhcb1 and P-Lhcb2 antibodies. Isolated thylakoids in either the dark- or the light-adapted state from wild type (WT) and PsbS-KO rice plants were used for this approach to detect light-dependent interactions between PsbS, PSII, and LHCII proteins. We observed that the bands corresponding to the phosphorylated Lhcb1 and Lhcb2 as well as the other phosphorylated proteins were enhanced in the PsbS-KO mutant after illumination. The qE relaxation became slower in WT plants after 10 min HL treatment, which correlated with Lhcb1 and Lhcb2 protein phosphorylation in the LHCII trimers under the same experimental conditions. Thus, we concluded that light-induced phosphorylation of PSII core and Lhcb1/Lhcb2 proteins is enhanced in rice PsbS-KO plants which might be due to more reactive-oxygen-species production in this mutant.

## 1. Introduction

Sunlight is the primary and key energy source for photosynthetic organisms; however, its quality and quantity are extremely variable in natural environments. Therefore, plants have to respond and acclimate to fluctuating light conditions by evolving a diversity of molecular regulatory mechanisms. These mechanisms control the photosynthetic apparatus’s structure and redistribute excitation energy between photosystems [1]. Diverse solutions to protect from excess light have evolved in vascular plants [2]. The molecular regulatory mechanisms that are involved in photoprotection of the photosynthetic apparatus are strictly interlinked within the thylakoid membranes of the photosynthetic organisms [3]. In vascular plants, the non-photochemical quenching (NPQ) of excess light energy protects against photodamage [4].

NPQ dissipates excess light energy by numerous diverse mechanisms [5,6,7]. Different components of NPQ emerge during illumination. The fast component of NPQ, energy-dependent quenching (qE), requires a trans-thylakoid proton gradient, zeaxanthin accumulation, and PsbS protein of photosystem (PS) II [8,9,10,11,12]. The slower components of NPQ are (1) qZ, zeaxanthin-dependent NPQ [13], (2) qT, state transition [14], (3) qI, photoinhibitory quenching [15], (4) qH, sustained quenching [6,16], and (5) qM, which is detected in the chloroplast movement mutant of Arabidopsis *phot2* [17] as a component lacking in the fluorescence decay [18].

Plants harmonize light harvesting, electron transport, and protein synthesis by adjusting the thylakoid membrane proteome in response to changes in the light environment [19,20]. The PsbS protein of PSII, responsible for qE, is also involved in the reorganization of the thylakoid membrane [21,22,23,24,25,26]. As a consequence, quenching may take place inside the light-harvesting complexes (LHC) by a conformational change of these proteins [27,28,29] and/or by the light-induced detachment of an antenna hetero-oligomer from PSII supercomplex [23]. While the active form of PsbS protein is likely monomeric, the non-activated form appears as dimers in the membrane. When dimeric PsbS protein is monomerized during acidification of the lumen [30,31,32] it induces a quenching state in the LHCII. The generation of NPQ during illumination is based on an enhanced interaction of PsbS with Lhcb1 [31]. Other interaction partners of PsbS protein, such as the minor LHCII antenna complex proteins, have also been suggested [32], although LHCII is expected to be the major player in NPQ [30]. During NPQ development, the thylakoid membrane of chloroplasts undergoes different ultrastructural changes. Such changes consist of loosening of the grana stacking [33], which allows spillover quenching of excess light energy via PSI [34,35]. The interaction of the stroma-exposed N termini of both the PSII and LHCII proteins of the polar thylakoids in grana influences grana stacking [36]. The thylakoid kinases STN7 and STN8 [37,38] regulate the state transitions [37] and also control the dimensions of the partition slit between polar thylakoid layers [39]. Reversible phosphorylation of the PSII and LHCII proteins by these kinases also regulates the structural protein CURVATURE THYLAKOID1 B (CURT1B) N terminus phosphorylation [33], the migration of PSs between grana stack and stroma thylakoid regions [40,41], the mixing of PSII–LHCII and PSI complexes in the grana margin regions [42], and the dynamic reorganization of the pigment–protein megacomplexes [43,44,45]. The phosphorylation of the LHCB1 isoform causes loosening of the thylakoid stacking in chloroplasts of vascular plants [33,46,47]. However, the thylakoid phosphoproteins of PSII and LHCII and their dynamics in the absence/presence of PsbS protein (during short and long illumination with low and high light) have not been well characterized.

Under low light intensity, dependent on the chromatic adaptation and metabolic conditions, the phosphorylated LHCII proteins become an efficient antenna for PSII and PSI which minimize NPQ [48]. Under varying light intensities, the protonation of the PsbS protein of PSII [22,23,25], and the phosphorylation of both the PSII core [49,50] and the LHCII proteins [51] facilitate the migration of the PSII-LHCII proteins along the thylakoid membrane. Also, PsbS-deficient Arabidopsis plants preserve higher LHCII phosphorylation levels under illumination than wild type and induce changes in the production of reactive oxygen species (ROS) [52]. We also observed higher ROS production in rice plants lacking the PsbS protein of PSII [10].

All these studies led to the suggestion that in vascular plants under natural environments, NPQ and the thylakoid protein phosphorylation work together in synchrony [45,48,53]. Therefore, here we investigated if the dynamics of thylakoid protein phosphorylation differ in the presence/absence of qE in rice plants under different light intensities. In this study, we used the rice wild-type (WT) and PsbS knock-out (Psbs-KO) plants for phosphoproteome investigations of the thylakoid protein complexes. We proposed that higher ROS production in the PsbS-KO mutant may enhance the phosphorylation of the PSII core and LHCII antenna proteins in the PsbS-KO plants after illumination.

## 2. Results

To identify the phosphorylation status of CP43, D2, D1, and light-harvesting complex (LHC) II proteins, the thylakoid membranes isolated from rice WT and mutant plants lacking PsbS protein of PSII (PsbS-KO) from dark- and light-adapted plant material were separated in SDS-PAGE and further immunodetected with P-Thr-specific antibody.

**Figure 1:**
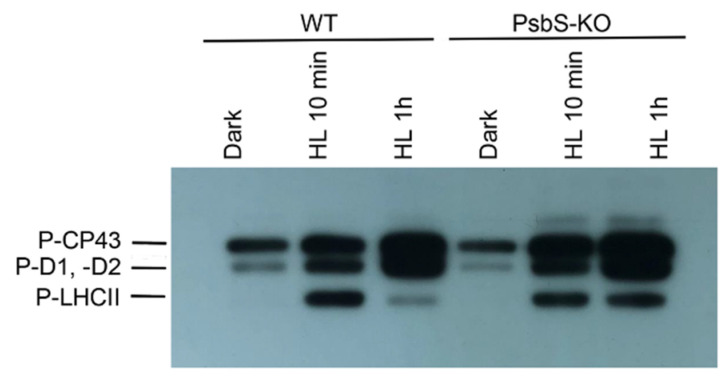
Immunoblot analysis of thylakoid membrane proteins separated by SDS-PAGE from wild-type (WT) and mutant (PsbS-KO) plants. The blots were incubated with phosphothreonine-specific antibodies (Anti-P-Thr, Agrisera AB, Vännas, Sweden). Thylakoids were isolated from the leaves dark-adapted for 12 h (Dark) and exposed to high light of 700 and 1500 µL photons m^−2^ s^−1^ for 10 min (HL 10 min) and 1 h (HL 1 h), respectively.

The phosphorylation levels of the major phosphoproteins of PSII core and LHCII were significantly affected by high light (HL) and by the loss of PsbS protein (Figure 1). In line with earlier studies, we observed the absence of the phosphorylated LHCII trimer after dark adaptation and the higher levels of the phosphorylated proteins after light treatments both in WT and PsbS-KO mutant lines. Strikingly, the phosphorylation level of LHCII was different between WT and PsbS-deficient mutant plants. The immunoblot revealed that the levels of the phosphorylated LHCII increased after 10 min light treatment in WT plants, whereas they were sharply reduced after 1 h light treatment. However, the levels of the phosphorylated LHCII remained stable during both light treatment conditions in the PsbS-KO mutant plants. The levels of the phosphorylated PSII core proteins were affected by the mutation and experimental conditions.

Thus, the PSII core phosphoprotein-band intensities (CP43, D1, and D2) from WT and PsbS-KO increased after 1 h HL treatment (Figure 1). The band intensities of the phosphorylated CP43, D1, and D2 proteins were higher in PsbS-KO plants in comparison with WT plants. In agreement with earlier reports [41], the phosphorylation level of CP43 was different from D1 and D2 proteins. The phosphorylation of D1 and/or D2 was lower in the mutant leaves both in darkness and light in contrast to the CP43 protein. Noteworthy, in the PsbS-KO mutant plants, the phosphorylation of the PSII core proteins was stronger when compared to WT. Furthermore, the amount of the phosphorylated CP43 was higher in dark-adapted WT plants; however, it reached the highest level in PsbS-KO mutants under both light treatment conditions (700 µL photons m^−2^ s^−1^ and 1500 µL photons m^−2^ s^−1^, respectively). Moreover, we detected one more band above the band of CP43 in PsbS-KO plants subjected to light exposure, and we propose that this band might be the phosphorylated CP47 protein of PSII. The phosphorylated CP47 protein of PSII was shown in samples of thylakoid membranes isolated from pine needles during cold acclimation [54]. Interestingly, we observed that LHCII was strongly phosphorylated after 10 min illumination in both genotypes while P-LHCII decreased over time (after 1 h illumination) in WT thylakoids. However, it still remained highly phosphorylated in thylakoids isolated from PsbS-KO leaves. Taken together, these data suggest that the lack of the PsbS protein in PsbS-KO rice mutants essentially stimulates the phosphorylation capability of thylakoids.

As a next step, to identify the protein subunits comprising the main thylakoid protein complexes (i.e., PSII, PSI, cytb_6_f, and ATP synthase) and their phosphorylation levels in the rice plant samples, we solubilized thylakoid membranes with a mild detergent (N-dodecyl β-D-maltoside (β-DM)), which allowed us to solubilize the thylakoid membranes and thus to resolve a different set of complexes [42,55]. The solubilized protein complexes were separated in their native state on the blue native polyacrylamide gel electrophoresis (BN-PAGE).

**Figure 2:**
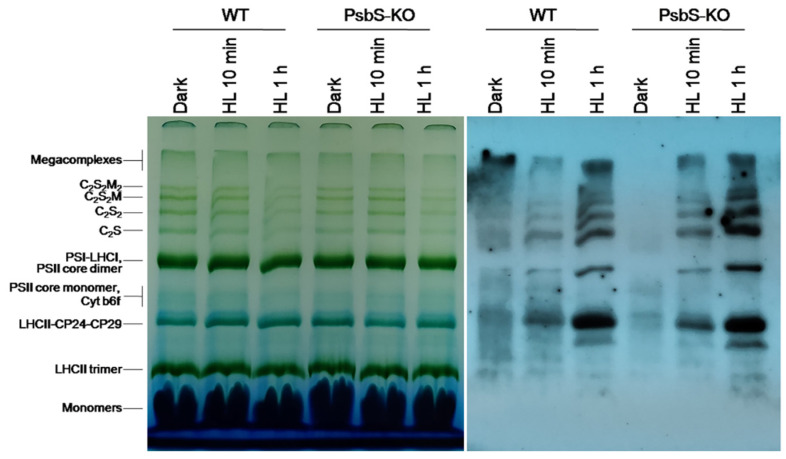
Immunolocalization of phosphoproteins in thylakoid membrane complexes of rice wild-type (WT) and PsbS knockout (PsbS-KO) mutant by using a phosphothreonine-specific antibody (Anti-P-Thr). Thylakoids were isolated from leaves dark-adapted for 12 h (Dark) and exposed to high light of 700 and 1500 µL photons m^−2^ s^−1^ for 10 min and 1 h, respectively, and separated by blue native (BN-PAGE) gel electrophoresis (**left** panel). For immunoblotting, the whole BN gels were electroblotted onto a polyvinylidene fluoride (PVDF) membrane using P-Thr antibody (**right** panel).

The result of the BN-PAGE showed only slight differences in the amount of the protein complexes including PSI/PSII, PSII core dimer, PSII core monomer, cytb6f, LHCII-CP24-CP29, LHCII trimer, and LHCII monomers in both WT and PsbS-KO plants under all experimental conditions (Figure 2). However, there were significant variations in the amount of the supercomplexes (SC) including, C_2_S_2_M_2_, C_2_S_2_M, C_2_S_2_, and C_2_S in both WT and PsbS-KO plants under different experimental conditions (Figure 2). High levels of SCs were detected in dark-adapted WT and PsbS-KO plants after 10 min illumination, while after 1 h illumination, the amount of the SCs was decreased in both WT and PsbS-KO plants. These results are in agreement with previous findings that SCs are degraded under high light [41]. To investigate the levels of the phosphorylated thylakoid membrane proteins, the resulted gels of the BN-PAGE were subjected to the direct transfer for immunoblotting of the thylakoid membrane proteins. Thus, the overall pattern of immunoblotting analysis of thylakoid membrane proteins separated by SDS-PAGE (Figure 1) and by BN-PAGE (Figure 2) from WT and PsbS-KO plants indicate that the levels of phosphorylation of thylakoid membrane proteins were higher in light-treated PsbS-KO samples than in the WT. The phosphorylation level in SCs showed a steady increase in the phosphorylation level of proteins after light exposure. Interestingly, in smaller SCs, the phosphorylation level was higher compared to larger SCs. Thus, the phosphorylation of supercomplex bands without M-trimer (C_2_S_2_ and C_2_S) was stronger than with M-timer (C_2_S_2_M and C_2_S_2_M_2_) both in WT and PsbS-KO samples. We propose that PSII-LHCII supercomplexes with high levels of LHCII phosphorylation may be less stable in vivo or may be labile during extraction. An alternative possibility is that, in the smaller PSII-LHCII complexes, LHCIIs may affect the phosphorylation of the PSII core, and/or Lhcb2 phosphorylation is more suppressed in the larger complexes [56,57].

Different brands and/or different batches of P-Thr antibodies reveal differences in specificity to distinct thylakoid phosphoproteins [58]. Therefore, to clarify LHCII phosphorylation dynamics during light treatment, we used the antibodies that detect the phosphorylated-lhcb1 (P-Lhcb1) and the phosphorylated-lhcb2 (P-Lhcb2). While carrying out quantitative studies on the kinetics and dynamics of the P-Lhcb1 and P-Lhcb2 in different supermolecular complexes and thylakoid domains is not a trivial undertaking by P-Thr, the P-Lhcb1 and P-Lhcb2 antibodies were used to overcome these limitations.

**Figure 3:**
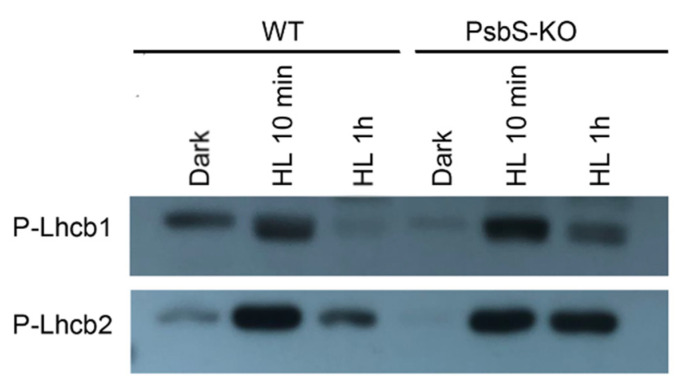
Detection of phosphorylated forms of Lhcb1 and Lhcb2 proteins in the thylakoid membranes separated by SDS-PAGE from rice wild-type (WT) and PsbS knockout (PsbS-KO) mutant plants with P-lhcb1 and P-lhcb2 antibodies. Thylakoids were isolated from the leaves dark-adapted for 12 h (Dark) and exposed to high light of 700 and 1500 µL photons m^−2^ s^−1^ for 10 min (HL 10 min) and 1 h (HL 1 h), respectively.

To study the light-dependent changes in the amount of P-Lhcb1 and P-Lhcb2, thylakoids isolated from the dark- and light-adapted plants of WT and PsbS-KO were subjected to SDS-PAGE and subsequent immunoblotting. As shown in Figure 3, we observed very low levels of P-Lhcb1 in the dark-adapted WT and PsbS-KO plants compared to light-treated samples; the levels of P-Lhcb1 increased in both genotypes after 10 min of light treatment (700 µL photons m^−2^ s^−1^). However, Lhcb1 was almost fully dephosphorylated in WT but remained weakly phosphorylated in PsbS-KO plants after 1 h light treatment (1500 µL photons m^−2^ s^−1^) (Figure 3). The overall pattern of P-Lhcb2 was similar to that observed for the P-Lhcb1. Nevertheless, some differences were also observed. We observed a very low-level P-Lhcb2 in the dark-adapted WT but in the samples of the PsbS-KO plants, P-Lhcb2 phosphorylation was not detected. On the other hand, upon 1 h illumination (1500 µL photons m^−2^ s^−1^), Lhcb2 became largely dephosphorylated in the WT but remained almost at the same phosphorylated level in the PsbS-KO plants (Figure 3). Interestingly, dark-adapted WT plants displayed some weak P-Lhcb1 and P-Lhcb2 bands, while PsbS-KO plants did not. Different phosphorylation patterns of the LHCII proteins in these two genotypes indicate that the phosphorylation of LHCII may play a role in the NPQ, especially in its energy-dependent part.

**Figure 4:**
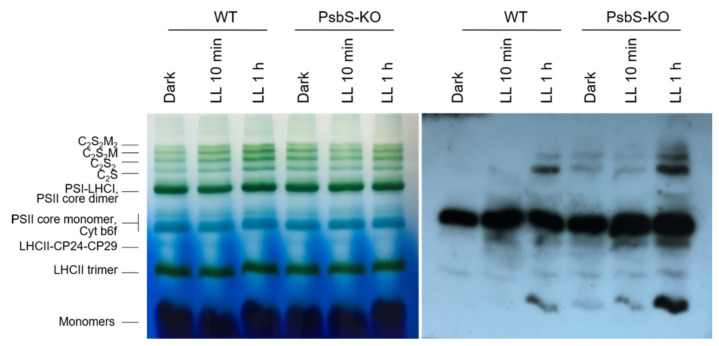
Immunolocalization of phosphoproteins in thylakoid membrane complexes of rice wild-type (WT) and PsbS knockout (PsbS-KO) mutant by using a phosphothreonine-specific antibody (Anti-P-Thr). Thylakoids were isolated from the leaves dark-adapted for 12 h (Dark), exposed to low light of 50 µL photons m^−2^ s^−1^ for 10 min (LL 10 min), and 1 h (LL 1 h) and separated by blue native (BN-PAGE) gel electrophoresis (**left** panel). For immunoblotting, the whole BN gels were electroblotted onto a polyvinylidene fluoride (PVDF) membrane (**right** panel).

The BN-PAGE experiment revealed significant variations in the distribution of the proteins in both the WT and PsbS-KO plants under different experimental conditions (Figure 4). There were differences in the amount of the supercomplexes (SC) including, C_2_S_2_M_2_, C_2_S_2_M, C_2_S_2_, and C_2_S in both WT and PsbS-KO plants under all experimental conditions (Figure 4). Higher levels of SCs were found in the WT in LL-adapted (1 h) plants, whereas the dark-adapted and 1 h LL-illuminated PsbS-KO plants contained similar high levels of SCs. WT showed a dramatically increasing tendency of band intensities from dark to 1 h low light exposure. When compared to WT, PsbS-KO displayed stronger bands of SCs in dark-adapted plants and relatively low intensity after 10 min of LL, whereas after 1 h of LL the band intensities of SCs increased again. In the case of bands belonging to PSII and LHCII, there were no visible differences.

The immunoblot analysis with P-Thr antibody demonstrated differences in the levels of phosphorylation of thylakoid membrane proteins of WT and PsbS-KO mutants (Figure 4). The phosphorylation levels of dark-adapted samples showed similar to LL 1 h-treated samples of WT with a relatively lower level of phosphorylation of C_2_S_2_ band and LHCII monomers. Plants treated with LL for 10 min demonstrated a lower level of phosphorylation of SCs and a higher level of phosphorylation of PSII core dimer and PSII-CP24-CP29 bands. However, plants treated with LL for 1 h demonstrated a higher level of phosphorylation of SCs and showed a steady increase in the phosphorylation levels of proteins after light exposure. A statistically significant level of the phosphorylated thylakoid membrane proteins was observed in the PsbS-KO rice plants (Figure 4). Interestingly, we observed phosphorylation of the LHC monomers in WT samples only exposed to LL for 1 h; however, LL-induced phosphorylation of the LHC monomers was detected in PsbS-KO samples. PsbS-KO LL 1 h-treated plants showed a strong increase in the phosphorylation levels of proteins in comparison with dark-adapted and 10 min-LL-exposed samples and also all WT samples.

**Figure 5:**
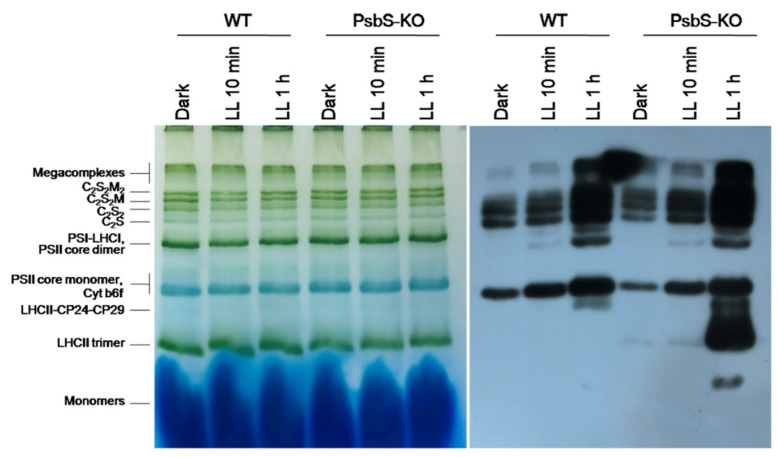
BN-PAGE and Western blot analysis of thylakoid membrane proteins extracted from leaves of rice wild-type (WT) and PsbS knockout (PsbS-KO) mutant—dark-adapted for 12 h (Dark) and exposed to low light of 50 µL photons m^−2^ s^−1^ for 10 min (LL 10 min) and 1 h (LL 1 h). Western blot analysis of leaves extracts with antibodies to P-lhcb1.

The immunoblot experiments revealed an increased level of phosphorylation of Lhcb1 after 1 h HL treatment in the supercomplexes and PSII core monomer (Figure 5); however, the phosphorylation levels of the bands related to the LHCII-CP29-CP24 bands in both WT and PsbS-KO genotypes appeared after 1 h low-light illumination. Interestingly, the phosphorylation of the LHCII trimer band appeared only in the PsbS-KO samples, and plants exposed to the LL for 1 h exhibited very high levels of the LHCII trimer band phosphorylation. Under the above experimental conditions, the phosphorylation of Lhcb1 was detected in the LHC monomer band. These data allow us to speculate that the strong LHCII trimer phosphorylation in PsbS-KO plants may compensate for the deficiency of PsbS protein in this plant. However, there is a possibility that this difference might be due to the different macroorganization of the thylakoid membrane in PsbS-KO plants. The Lhcb1 phosphorylation pattern of the PSI-LHCI/PSII dimer bands is (Figure 5) also similar to the data obtained with high light intensities.

**Figure 6:**
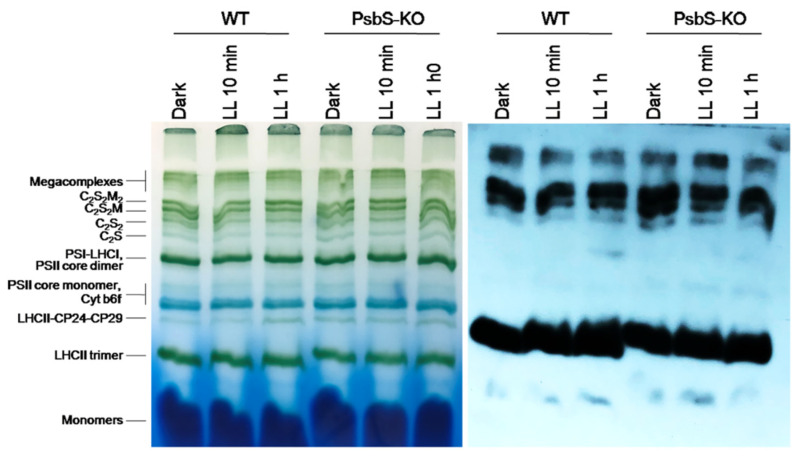
BN-PAGE and Western blot analysis of thylakoid proteins extracted from leaves of rice wild-type (WT) and PsbS knockout (PsbS-KO) mutant dark-adapted for 12 h (Dark) and exposed to low light of 50 µL photons m^−2^ s^−1^ for 10 min (LL 10 min) and 1 h (LL 1 h). Western blot analysis of leaves extracts with antibodies to Lhcb2.

As shown in Figure 6, there were no differences in the Lhc2 phosphorylation pattern in all experimental conditions. Thus, we speculate that P-Lhcb1 may play some compensative role due to the lack of the PsbS protein in the PsbS-KO plants. Next, we investigated whether NPQ induction and relaxation kinetics were correlating with protein phosphorylation patterns in both examined WT and PsbS-KO genotypes under the same experimental conditions.

**Figure 7:**
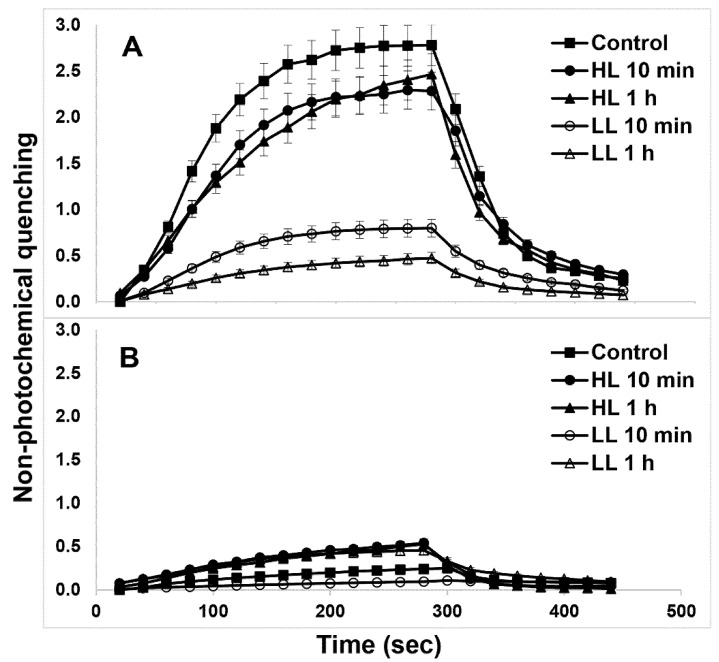
NPQ development in plants subjected to the different light treatment conditions. Wild type (**A**) and PsbS-KO (**B**). Leaves of plants were dark-adapted for 10 min (Dark) before measurements. The actinic light intensity used for NPQ measurements was 700 µL photons m^−2^ s^−1^. Values are means ± SD (*n* = 5).

The NPQ induction and relaxation kinetics in both WT (Figure 7A) and PsbS-KO (Figure 7B) rice genotypes under both LL- and HL-treatment states are shown in Figure 7. HL-treated WT plants showed a larger magnitude of NPQ after 5 min illumination relative to their LL-treated counterparts (Figure 7A). PsbS-KO plants also showed a similar tendency but with much a smaller magnitude (Figure 7B). Although the differences in NPQ were much more significant between the WT and the PsbS-KO mutant of rice plants, HL-treated both WT and PsbS-KO plants had higher NPQ than LL-treated plants; under the same experimental conditions, they also displayed higher Lhcb1 and Lhcb2 protein phosphorylation in LHCII trimer than their LL-treated counterparts.

Because it is assumed that plants with high NPQ capacity can re-establish high rates of photosynthesis quickly [59]. the NPQ dark relaxation kinetics of the investigated samples were fitted to the double exponential model [60]. There was a change in qE relaxation (τ_1_) and qZ phase of NPQ relaxation (τ_2_) in both genotypes after subsequent light treatments (Table 1). Interestingly, the qE relaxation (τ_1_) became slower in WT plants after 10 min HL treatment than under any other experimental condition. These data allow us to speculate that the phosphorylation of Lhcb1 and Lhcb2 proteins are closely related to the quick NPQ relaxation as well as to the high rate of photosynthesis.

## 3. Discussion

The qE component of NPQ and especially the PsbS protein of PSII is a vital factor that regulates the dynamic response of the thylakoid membrane to fluctuating light intensities. Nevertheless, its molecular mechanisms are not well known as, at the same time, the other components of NPQ such as qZ [13], qT [14], qI, [15], qH [6,16], and qM [18] may be simultaneously involved. Recently published works [45,48,53] suggest that thylakoid protein phosphorylation affects the NPQ processes. In addition, the absence of the PsbS protein enhances the light-induced thylakoid protein phosphorylation [52]. However, it is still unclear exactly what kind of thylakoid proteins and their phosphorylated forms as well as what dynamic changes in the thylakoid protein complexes might interact during NPQ generation. Here, we have established a direct Western blot assay after BN-PAGE that allowed us to detect all phosphoproteins by P-Thr antibody as well as P-Lhcb1 and P-Lhcb2 proteins of isolated thylakoids in either the dark- or the light-adapted states. To the best of our knowledge, this approach was used for the first time to detect the dependence of phosphorylation of Lhcb1, Lhcb2, and other phosphoproteins of thylakoid membranes on the presence/absence of PsbS, under different illumination conditions of rice plants.

We have previously shown that the absence of the PsbS protein does not significantly affect the zeaxanthin accumulation and the state transition [26]. On the other hand, there were differences between WT and PsbS-KO plants in the PSII repair system [10], the cyclic electron flow around PSI [61], the resistance to bacterial and fungal pathogens [62], and molecular oxygen dependency of NPQ [7]. Therefore, we assume that the qZ, qT, and qM components of the NPQ were negligible under our experimental conditions. To consider the role of qI, we used high light intensity (1500 μL photons m^−2^ s^−1^) for 1 h. Thylakoid protein phosphorylation has been related to sustained NPQ [63], but we assume that during a short time (10 min of illumination) qH is also negligible.

The major LHCII of land plants consists of three different Lhcb proteins (Lhcb1-3). Our data reveal the principal interaction of PsbS with PSII core and LHCII phosphoproteins in the dark-adapted state and enhanced interaction with P-Lhcb1 in the qE-active light state. It is well known that PsbS-dependent qE is a very rapid process, taking place in seconds to minutes, however, in most of our experiments, the major alterations in phosphorylation were observed after 1 h. Thus we cannot exclude the possibility of the phosphorylation response to the impaired qE. There is evidence that at least part of the increased phosphorylation in light-treated PsbS-KO is in the PSII core complex (Figure 1). This would not be surprising if we consider that (1) the absence of PsbS-induced qE intensifies the excitation pressure and stress in HL and (2) STN8-dependent phosphorylation of PSII core proteins is involved in the PSII repair under photoinhibitory conditions [45]. Fascinatingly, it has been shown that Lhcb1 is highly phosphorylated in the PSII–LHCII supercomplexes, while Lhcb2 is highly phosphorylated in the disconnected L-LHCII trimer [63], which is in agreement with our results. In the case of qI, phosphorylation of the PSII and Lhcb2 proteins appears to be more important. It has been shown that the absence of Lhcb3, which is not phosphorylated, *Arabidopsis thaliana* T-DNA knockout plants lacking Lhcb3 (koLhcb3) do not display a noteworthy alteration in PSII efficiency and/or qE type of NPQ, but the rate of transition from state 1 to state 2, increases in koLhcb3, suggesting that Lhcb3 modulates the rate of state transitions [64]. Unfortunately, we could not detect Lhcb3 phosphorylation in our work. Intriguingly, while the LHCII trimer band showed low Lhcb1 phosphorylation in dark-adapted samples of both WT and PsbS-KO mutant, it was highly phosphorylated after 10 min of light treatment (700 µL photons m^−2^ s^−1^) and almost completely dephosphorylated after 1 h of the same light-intensity treatment. While there was no detected phosphorylated Lhcb2 band in LHCII trimer from dark-adapted samples, after 10 min of light treatment, the LHCII trimer was highly enriched with Lhcb2 spots. It was reported that strong Lhcb2 phosphorylation in L-LHCII trimers is a prerequisite for energy transfer from LHCII to PSI [65,66]. Compared to WT, PsbS-KO showed lower phosphorylation levels of P-Lhcb1 and P-Lhcb2 in the LHCII trimer band both in dark and HL treated samples. HL-treated plants displayed slower qE relaxation kinetics (Table 1) than their LL-treated counterparts and also displayed higher Lhcb1 and Lhcb2 protein phosphorylation in LHCII trimer than their LL-treated counterparts (Figure 3 and Figure 5).

In addition, phosphorylation of CP29 and NPQ are two processes which are considered to be independent of one another. However, a connection between NPQ and phosphorylation of CP29 in monocots was also shown [67]; the authors proposed that phosphorylation causes a conformational change of CP29 in rice plants which disturbs the conformation of the whole PSII supercomplex. This idea was proved by the partial disassembly of PSII-LHCII supercomplex into smaller complexes [67]. Because phosphorylation of PSII core subunits and CP29 obligingly yields the increased photoprotection phenotype, our more detailed investigation of PSII core phosphorylation confirmed this idea. Thus, most of our results clearly indicate that phosphorylation of both PSII core and LHCII proteins is enhanced in the PsbS-KO rice mutant after illumination compared to WT plants.

Altogether, our data allow us to conclude that Lhcb1 and Lhcb2 phosphorylation is somehow involved in qE relaxation kinetics. On the other hand, based on the accumulation of P-Lhcb1 in SCs in HL 1 h-treated PsbS-KO leaves and in both SC and LHCII trimers and monomers in LL 1 h-treated PsbS-KO leaves, we can suggest that some defects shown in PsbS-KO leaves may inhibit the dephosphorylation of Lhcb1. A candidate for this triggering effect would be more ROS generated in the PsbS-KO leaves under high light conditions [10]. However, we also cannot exclude the other possibilities because the absence of PsbS may influence many other features of the thylakoids of rice plants [7,10,61], and thus, phosphorylation might be just another. The phosphorylation of the PsbS protein in spruce suggests an essential role for the p-PsbS protein in sustained quenching [63]. This might be true also for qE. The dynamic functioning of the plant thylakoid membrane upon changing light intensity depends on PsbS interactions with the LHCII and PSII core proteins as well as with their phosphorylation. We may consider that the light intensity through the redox state of the plastoquinone pool controls the functions of the PsbS protein and STN kinases. Moreover, as Aro’s group suggested, the functions of the STN7 and STN8 kinases are entirely coordinated with the PsbS [48]. Thus, to survive under changing light and/or other environmental conditions, plants need the harmonized action of the NPQ (especially qE), together with the reversible phosphorylation of the PSII core and antenna proteins.

## 4. Materials and Methods

### 4.1. Plant Materials and Light Treatment

Rice (*Oryza sativa* L.) WT Hwayoung cultivars and PsbS-KO mutant were grown in a greenhouse at 30/26 °C (day/night), with a light photoperiod of 16 h of light/8 h of dark. The middle parts of the leaves of one-month-old seedlings were used for experiments.

To analyze light-dependent changes of thylakoid membrane proteins, WT and PsbS-deficient mutant plants were dark-adapted overnight (12 h) before being exposed to light of different intensities (LL: 50 µL photons m^−2^ s^−1^ for 10 min or 1 h; HL: 700 µL photons m^−2^ s^−1^ for 10 min and 1500 µL photons m^−2^ s^−1^ for 1 h).

### 4.2. Isolation of Thylakoid Membranes and Chlorophyll Determination

Thylakoid membranes were isolated from one-month-old rice leaves as described by [54] with some modifications. Leaves for experimental samples were frozen directly under light conditions to escape quick dark relaxation processes. Frozen leaves were pulverized in liquid nitrogen and then ground in ice-cold grinding buffer (50 mM Hepes/KOH (pH 7.5), 330 mM sorbitol, 2 mM EDTA, 1 mM MgCl_2_, 5 mM ascorbate, 0.05% BSA, and 10 mM sodium fluoride). The homogenate was filtered through four layers of Miracloth (Merck, Darmstadt, Germany) followed by centrifugation at 5500× *g* for 6 min at 4 °C. The resulting pellet was washed and suspended in a shocking buffer (50 mM Hepes/KOH (pH 7.5), 5 mM sorbitol, 5 mM MgCl_2_, and 10 mM NaF) and then centrifuged at 5500× *g* for 6 min at 4 °C. The pellet was resuspended in a storage buffer (50 mM Hepes/KOH (pH 7.5), 100 mM sorbitol, 10 mM MgCl_2_, and 10 mM NaF) followed by centrifugation at 5500× *g* for 6 min at 4 °C. Finally, the thylakoid pellet was suspended into a small aliquot of storage buffer. All the stages in thylakoid preparation were performed on ice under dim light. The chlorophyll concentration was determined from the samples as described in [68].

### 4.3. Blue-Native Polyacrylamide Gel Electrophoresis

The BN-PAGE was performed according to [55] with some modifications. Thylakoid membranes with 8 µg chlorophyll were pelleted at 5500× *g* for 6 min at 4 °C. The resulting pellet was resuspended into 10 µL ice-cold buffer containing 25 mM BisTris/HCl (pH 7.0), 20% (*w*/*v*) glycerol, 10 mM NaF, and 0.25 mg ml^−1^ Pefabloc. An equal volume of detergent solution (which was diluted in this buffer) was added to a final concentration of 1.0% (*w*/*v*) β-DM (Sigma, St. Louis, MO, USA). The thylakoid membranes were incubated in darkness for 5 min on ice; insolubilized material was removed by centrifugation at 13,000 rpm for 10 min at 4 °C. Prior to loading, the samples were supplemented with a one-tenth volume of Serva Blue G buffer (100 mM BisTris/HCl (pH 7.0), 0.5 M amino-n-caproic acid, and 0.25 mg ml^−1^ Pefabloc, 30% (*w*/*v*) sucrose, and 50 mg·ml^−1^ Serva Blue G) to introduce a negative charge and to increase the solubility of the sample. BN-PAGE was performed with a linear gradient of 4.5–13.5% separation gel and 4% (*w*/*v*) stacking gel [69]. The samples were run by 10 mA (~100–125 V) at 4 °C for 35–40 min with cathode buffer (50 mM tricine, 15 mM BisTris) including 0.01% (*w*/*v*) Serva Blue G stain (Coomassie) and, as the blue running front had moved about a half in the cathode, a buffer without Coomassie. The BN-PAGE was stopped when the Coomassie blue ran out of the bottom of the gel. The same anode buffer (50 mM BisTris/HCl (pH 7.0)) was used during all native PAGE.

### 4.4. Sodium Dodecyl Sulfate–Polyacrylamide Gel Electrophoresis and Immunoblotting Analysis

Thylakoid membranes were isolated from dark-adapted (12 h darkness) and light-adapted (10 min at 700 µL photons m^−2^ s^−1^, 1 h at 1500 µL photons m^−2^ s^−1^) rice WT and mutant plants lacking PsbS protein of PSII according to [70]. Thylakoid membrane proteins were separated in SDS-PAGE, and the phosphorylation levels of CP43, D2, D1, and light-harvesting complex (LHC) II were visualized by immunodetection with P-Thr-specific antibody. Sodium dodecyl sulfate–polyacrylamide gel electrophoresis (SDS-PAGE) was performed as described by [71]. Thylakoid membranes with 2 µg of chlorophylls were solubilized with Laemmli buffer and separated in SDS-PAGE using the tris-glycine system at 12% acrylamide mix concentration. Thylakoid membrane proteins separated by SDS-PAGE were transferred to polyvinylidene fluoride (PVDF) membrane (Immobilon-P, Merck, Darmstadt, Germany). Immunoblot analysis was performed as described by [72], with P-Thr, P-Lhcb1, and P-Lhcb2 antibodies (Agrisera AB, Vännas, Sweden).

Direct immunoblotting from BN-PAGE was performed as described by [73]. The whole BN gels were incubated in SDS sample buffer (100 mM Tris-HCl, pH 6.8, 2% SDS, 15% glycerol) for 1 h with gentle shaking and subsequently electroblotted onto polyvinylidene fluoride (PVDF) membranes and immunoblotted with specific antibodies: anti-P-Thr, P-Lhcb1, and P-Lhcb2. Development was performed with an electrochemiluminescence detection system, exposing the blots to the X-ray film with different exposure times.

### 4.5. Chlorophyll Fluorescence and Electron Transport Measurements

The Fv/Fm ratio was measured in detached leaves with a PAM fluorometer (PAM2000, Walz, Effeltrich, Germany) using an actinic light intensity of 700 µL photons m^−2^ s^−1^ and at 22–25 °C, as described previously [7]. The latest research highlights that Fv/Fm ratio cannot be equated with the photochemical efficiency of PSII [74]. The NPQ values were determined from (Fm−Fmʹ)/Fmʹ, where Fm is the maximum yield of chlorophyll-a fluorescence measured in dark-acclimated leaves, while Fmʹ is the maximum yield measured in light-acclimated leaves. NPQ relaxation kinetics was analyzed according to [60].

### 4.6. Statistical Analysis

Microsoft Excel software (Microsoft Office 2016 for Windows) was used for statistical analyses (one-way ANOVA). All BN-PAGE and Western blot images were digitized using ImageJ software. Data are stated as mean values ± standard deviation. The *t*-test was employed to define the statistical significance (*p* < 0.01 with one asterisk and *p* < 0.05 with two asterisks) of the alterations between the means. The number of biological and analytical replicates was at least three. All digitized data shown as Supplementary Tables S1–S6 for Figure 1, Figure 2, Figure 3, Figure 4, Figure 5 and Figure 6.

## 5. Conclusions

NPQ and thylakoid protein phosphorylation are two important mechanisms that protect vascular plants. Therefore, the detailed characterization of these processes using plants lacking the energy-dependent (qE) part of NPQ is important for a reliable identification of functionally relevant phosphoproteins during quenching processes. Although it is clear that NPQ and the thylakoid protein phosphorylation work together in synchrony, their molecular mechanism is still unclear. Thus, the identification of thylakoid protein phosphorylation during quenching processes will help to better understand the underlying molecular mechanisms. Furthermore, analysis of the phosphorylated proteins will help to detect novel marker candidates for NPQ and the origin of their action. The reorganization of plant thylakoid membranes during light intensity changes depends on PsbS interactions with the LHCII and PSII core proteins as well as with their phosphorylation. Therefore, we conclude that more ROS production in rice PsbS-KO may cause enhanced light-induced phosphorylation of PSII core and antenna proteins.

## Figures and Tables

**Table 1:** Analysis of decay time constant (τ_i_, s), amplitude (A_i_, arbitrary units), and significance level (R) of NPQ relaxation kinetics after actinic light is turned off. The traces shown in Figure 7 were used for the analysis.

Sample		R	τ_1_	A_1_	τ_2_	A_2_
Wild type	Control	0.997 ± 0.001	25.2 ± 2.1	2.51 ± 0.11	1577 ± 145	0.64 ± 0.08
	10 min HL	0.988 ± 0.001	53.9 ± 6.4	2.19 ± 0.09	51,853 ± 682	0.39 ± 0.05
	1 h HL	0.996 ± 0.001	33.1 ± 2.7	1.98 ± 0.12	326 ± 24	0.69 ± 0.09
	10 min LL	0.997 ± 0.001	30.7 ± 3.2	0.47 ± 0.08	3344 ± 287	4.29 ± 0.21
	1 h LL	0.997 ± 0.001	33.1 ± 4.1	0.35 ± 0.09	1149 ± 98	0.43 ± 0.11
PsbS-KO	Control	0.998 ± 0.001	21.8 ± 2.2	0.16 ± 0.05	2345 ± 186	0.29 ± 0.04
	10 min HL	0.997 ± 0.001	24.8 ± 2.7	0.51 ± 0.07	*n*.d.	0.02 ± 0.00
	1 h HL	0.994 ± 0.001	36.5 ± 4.4	0.60 ± 0.09	52.11 ± 0.19	0.002 ± 0.00
	10 min LL	0.953 ± 0.001	37.5 ± 3.6	0.04 ± 0.03	*n*.d.	0.004 ± 0.00
	1 h LL	0.996 ± 0.001	23.7 ± 2.5	0.06 ± 0.03	49.39 ± 0.21	0.31 ± 0.03

The data were fitted to a two-component exponential decay equation without normalization.

## Supplementary Materials

The following are available online at https://www.mdpi.com/article/10.3390/ijms22157978/s1.
