# Supplementary material for: Role of Thylakoid Protein Phosphorylation in Energy-Dependent Quenching of Chlorophyll Fluorescence in Rice Plants
Source: Int J Mol Sci. 2021 Jul 26;22(15):7978. doi: 10.3390/ijms22157978 (PMC8347447; doi:10.3390/ijms22157978)
Supplement: Supplementary file 1 [file ijms-22-07978-s001.zip › ijms-1263449-supplementary.pdf]

Role of Thylakoid Protein Phosphorylation in Energy-Dependent Quenching of Chlorophyll Fluorescence in Rice Plants

Aynura Pashayeva, Guangxi Wu, Irada Huseynova, Choon-Hwan Lee, Ismayil S. Zulfugarov

Supplementary Tables S1~S6 for Figures 1~6

Supplementary Table S1 for Figure 1

	WT			PsbS-KO		
**Bands**	**Dark**	**HL 10 min**	**HL 1 h**	**Dark**	**HL 10 min**	**HL 1 h**
**P-CP47**	0	0	0	0	1485±76	1621±92
**P-CP43**	5446±354	8516±452**	8716±481**	4141±203	9474±468**	10618±595**
**P-D1/D2**	2788±182	5895±313**	7549±425**	1640±85	5680±274**	9802±541**
**P-LHCII**	0	6614±351	2727±155**	0	5130±256	5320±294

Supplementary Table S2a for Figure 2a

	WT			PsbS-KO		
**Bands**	**Dark**	**HL 10 min**	**HL 1 h**	**Dark**	**HL 10 min**	**HL 1 h**
**MC**	2017±163	2424±191*	2105±158	2352±162	1852±143*	2630±203*
**$C_2S_2M_2$**	1958±159	1877±148	2205±165*	1918±132	1847±142	1092±84**
**C_2S_2M**	2887±234	2357±186*	1750±131**	2110±146	2308±178*	914±70*
**C_2S_2**	2879±233	2508±198*	1517±114**	2472±171	2410±186	1935±149**
**C_2S**	3167±257	3441±272*	2273±170**	2833±195	2574±198*	1571±121**
**PSI/PSII**	9955±806	11223±887*	10929±820	9940±686	9412±725	8522±656*
**PSII**	4305±349	3406±269*	2566±192**	2717±187	2572±198*	1620±125**
	0	0	0	0	0	0
**CP24-CP29**	5320±431	8382±662**	8278±621**	6214±429	5436±419*	7693±592*
**LHCII trimer**	11225±909	10783±852	9883±741	10511±725	10522±810	10554±813
**LHCII monomer**	122995±885	13172±948	13952±1005	13755±990	15265±1099	16215±1161

MC – Megacomplexes; PSI/PSII – PSI-LHCI/PSII core dimer; PSII – PSII core monomer/$Cytb_6f$; CP24-CP29 – PSII-CP24-CP29.

Supplementary Table S2b for Figure 2b

	WT			PsbS-KO		
**Bands**	**Dark**	**HL 10 min**	**HL 1 h**	**Dark**	**HL 10 min**	**HL 1 h**
**MC**	8828±758	2760±351**	6171±666*		3293±353*	5309±596*
**$C_2S_2M_2$**						
**C_2S_2M**		1957±188*	3415±114*		2278±197*	4364±499*
**C_2S_2**		2514±234	3495±322		2759±299*	3761±394*
**C_2S**	2663±255	3067±375	4168±386**		4426±468*	5513±488*
**PSI/PSII**		2793±272	3619±334		2285±267*	4681±519*
**PSII**		3289±383*		2115±270		
		6687±656*	10665±933*	2643±255	4560±433**	9274±822*
**CP24-CP29**	6513±586		2871±294*			2783±285*
**LHCII trimer**	2423±264					1595±114*
**LHCII monomer**						

MC – Megacomplexes; PSI/PSII – PSI-LHCI/PSII core dimer; PSII – PSII core monomer/Cytb_6f; CP24-CP29 – PSII-CP24-CP29.

Supplementary Table S3 for Figure 3

	WT			PsbS-KO		
**Bands**	**Dark**	**HL 10 min**	**HL 1 h**	**Dark**	**HL 10 min**	**HL 1 h**
**P-Lhcb1**	5318±511	8581±824**	1812±174**	2868±275	9776±938**	6900±662**
**P-Lhcb2**	3829±369	9477±912**	6039±580**	547±33	9264±889**	8289±796*

Supplementary Table S4a for Figure 4a

	WT			PsbS-KO		
**Bands**	**Dark**	**LL 10 min**	**LL 1 h**	**Dark**	**LL 10 min**	**LL 1 h**
**$C_2S_2M_2$**	469±31	737±49*	849±56*	595±39	603±40	793±52*
**C_2S_2M**	780±51	1133±95**	1553±102**	976±64	909±93	1610±108**
**C_2S_2**	839±55	1337±51**	1575±114**	1237±82	1079±97*	1805±119**
**C_2S**	969±64	1088±73	1222±81*	1248±82	1179±78	1420±94*
**PSI/PSII**	3868±255	3381±223	3829±253	4080±269	3616±239*	3912±258
**PSII**	1731±114	1512±113*	1127±74**	1327±88	1043±69*	813±54**
	3560±235	3810±251*	3623±239	3993±264	3736±267*	4361±288**
**CP24-CP29**	530±35	522±34	505±33	521±34	514±36	509±37
**LHCII trimer**	6804±449	5609±370	5814±384	6045±399	6239±412	6366±474
**LHCII monomer**	24905±1644	18456±1218*	22103±1459	22179±1464	21025±1388	24162±1595

PSI/PSII – PSI-LHCI/PSII core dimer; PSII – PSII core monomer/Cytb$_6$f; CP24-CP29 – PSII-CP24-CP29.

Supplementary Table S4b for Figure 4b

	WT			PsbS-KO		
**Bands**	**Dark**	**LL 10 min**	**LL 1 h**	**Dark**	**LL 10 min**	**LL 1 h**
**$C_2S_2M_2$**	0	0	0	0	0	0
**C_2S_2M**	0	0	0	0	0	494±39
**C_2S_2**	0	0	1287±102	1047±83	342±27**	3065±242**
**C_2S**	0	0	4749±375	1326±105	980±77*	6404±506**
**PSI/PSII**	0	0	0	0	0	0
**PSII**	0	0	0	0	0	0
	12392±979	14629±1156	15170±1198*	14463±1143	16432±1298	17781±1405*
**CP24-CP29**	0	1665±132	1116±88*	921±73	1846±146*	4171±330**
**LHCII trimer**	0	0	0	0	0	
	939±74	506±40*	860±68	873±69	1540±122*	2052±162*
**LHCII monomer**	0	0	2477±196	1343±106	2342±185*	9595±758**

PSI/PSII – PSI-LHCI/PSII core dimer; PSII – PSII core monomer/Cytb$_6$f; CP24-CP29 – PSII-CP24-CP29.

Supplementary Table S5a for Figure 5a

	WT			PsbS-KO		
**Bands**	**Dark**	**LL 10 min**	**LL 1 h**	**Dark**	**LL 10 min**	**LL 1 h**
**MC**	2243±132	1941±111	2111±152	2163±149	19700±156	2406±166*
**$C_2S_2M_2$**	1267±75	743±42*	1280±92	1448±100	1195±94*	1179±81*
**C_2S_2M**	1317±78	899±51*	1249±90	1483±102	1228±97	1305±90
**C_2S_2**	1087±64	578±33*	982±71	1077±74	956±76	923±64
**C_2S**	844±50	798±45	678±49	738±51	737±58	612±42
**PSI/PSII**	152±9	162±9	138±10	177±12	191±15	311±21*
**PSII**	3219±190	2170±124*	2432±175*	2885±199	3142±248*	3067±212
	493±29	166±9*	238±17*	255±18	271±21	198±14
**CP24-CP29**	2469±146	2582±147	3266±235*	3239±223	3364±266	3968±274*
**LHCII trimer**	3557±210	3614±206	3790±273*	3959±273	3696±292	3878±261
**LHCII monomer**	19278±1137	21147±1205	19435±1399	22094±1524	20157±1592	20607±1422

MC – Megacomplexes; PSI/PSII – PSI-LHCI/PSII core dimer; PSII – PSII core monomer/Cytb$_6$f; CP24-CP29 – PSII-CP24-CP29.

Supplementary Table S5b for Figure 5b

	WT			PsbS-KO		
**Bands**	**Dark**	**LL 10 min**	**LL 1 h**	**Dark**	**LL 10 min**	**LL 1 h**
**MC**	1380±95	1801±135*	7660±544**	2050±139	3272±226*	8926±696**
**$C_2S_2M_2$**	0	0	4734±341	0	0	oversaturated
**C_2S_2M**	2124±147	2048±154	4729±340**	3545±241	5530±382**	oversaturated
**C_2S_2**	4490±310	3660±275	4840±348	3810±259	6273±433**	oversaturated
**C_2S**	3996±276	3408±323	5132±370*	2872±195	4991±344*	oversaturated
**PSI/PSII**	124±9	166±12	1606±116**	0	0	0
**PSII**	0	767±58	3523±254**	0	1029±71	3415±266**
	0	0	0	0	0	8270±645
**CP24-CP29**	5738±396	8106±608**	9922±714**	3842±261	7089±489**	2385±186*
**LHCII trimer**	0	0	1048±75	606±41	979±68*	18251±1424* *
**LHCII monomer**	0	0	0	0	0	2776±217

MC – Megacomplexes; PSI/PSII – PSI-LHCI/PSII core dimer; PSII – PSII core monomer/Cytb$_6$f; CP24-CP29 – PSII-CP24-CP29.

Supplementary Table S6a for Figure 6a

	WT			PsbS-KO		
**Bands**	**Dark**	**LL 10 min**	**LL 1 h**	**Dark**	**LL 10 min**	**LL 1 h**
**MC**	680±40	778±51	956±66*	622±39	930±63*	1156±84*
**$C_2S_2M_2$**	594±35	480±31	1080±75**	546±34	653±44	593±43
**C_2S_2M**	724±43	940±52*	1303±90**	467±29	1302±89**	1309±96**
**C_2S_2**	1022±60	804±351*	1072±74	577±36	1148±78**	1022±75**
**C_2S**	648±38	623±40	725±50*	588±36	776±53*	546±40
**PSI/PSII**	292±17	169±11	261±18	580±36	457±31	397±29
**PSII**	2684±158	2109±137*	3035±209*	2343±145	3154±214*	3018±220*
	265±16	339±2*2	291±20	389±24	565±38*	328±24
**CP24-CP29**	2950±174	2757±179	2714±187	3189±198	3325±226	3524±257*
**LHCII trimer**	360±21	461±30	999±69*	656±41	761±52	829±61*
**LHCII monomer**	3352±198	2636±171*	3179±219	2667±165	3169±215*	2677±195
**MC**	19652±1159	17217±1119	15305±1056	20001±1240	16264±1106	16598±1212

MC – Megacomplexes; PSI/PSII – PSI-LHCI/PSII core dimer; PSII – PSII core monomer/Cytb$_6$f; CP24-CP29 – PSII-CP24-CP29.

Supplementary Table S6b for Figure 6b

	WT			PsbS-KO		
**Bands**	**Dark**	**LL 10 min**	**LL 1 h**	**Dark**	**LL 10 min**	**LL 1 h**
**MC**	0	0	0	0	0	
**$C_2S_2M_2$**	3247±237	3086±235	2987±242	4176±276	4996±345	3077±228*
**C_2S_2M**						
**C_2S_2**	7643±558	6689±508	8641±700	7494±495	6196±428*	6778±502
**C_2S**	4207±307	3882±295	3701±300*	6677±441	3891±268*	4038±299*
**PSI/PSII**	555±41	738±56*	948±7*7	781±52	957±66	423±31*
**PSII**						
			518±42			
**CP24-CP29**					290±20	290±21
**LHCII trimer**						
**LHCII monomer**	14175±1035	16358±1243	19414±1573*	14433±953	15806±1091	`16662±1233*
	414±30	752±57*	991±80*	339±22	1201±83**	292±22

MC – Megacomplexes; PSI/PSII – PSI-LHCI/PSII core dimer; PSII – PSII core monomer/Cytb$_6$f; CP24-CP29 – PSII-CP24-CP29.
